# The Decreased Growth Hormone Response to Growth Hormone Releasing Hormone in Obesity Is Associated to Cardiometabolic Risk Factors

**DOI:** 10.1155/2010/434562

**Published:** 2010-01-21

**Authors:** Fernando Cordido, Jesús Garcia-Buela, Susana Sangiao-Alvarellos, Teresa Martinez, Ovidio Vidal

**Affiliations:** ^1^Department of Endocrinology, Hospital A Coruña, 15006 A Coruña, Spain; ^2^Department of Investigation, Hospital A Coruña, 15006 A Coruña, Spain; ^3^Department of Medicine, University of A Coruña, 15006 A Coruña, Spain; ^4^Department of Laboratory, Hospital A Coruña, 15006 A Coruña, Spain

## Abstract

The aim of the present study was to evaluate the relationship between GHRH-induced GH secretion in obese premenopausal women and cardiovascular risk markers or insulin resistance. Premenopausal obese women, aged 35–52 years, were studied. GH secretion, IGF-I, serum cardiovascular risk markers, insulin, leptin, mid-waist and hip circumference, total body fat, and truncal fat were measured. Subjects were classified as meeting the criteria for GH deficiency (GHD) when peak GH after stimulation with GHRH was ≤3 *μ*g/L. Mean total and LDL cholesterol, fasting insulin, and HOMA-IR were all higher, in subjects who would have been classified as GH-deficient compared with GH-sufficient. Peak GH secretion after stimulation was inversely associated with fasting insulin (*R* = −0.650, *P* = .012), HOMA-IR (*R* = −0.846, *P* = .001), total cholesterol (*R* = −0.532, *P* = .034), and LDL cholesterol (*R* = −0.692, *P* = .006) and positively associated with HDL cholesterol (*R* = 0.561, *P* = .037). These data strongly suggest a role for insulin resistance in the decreased GH secretion of obesity and that the blunted GH secretion of central obesity could be the pituitary expression of the metabolic syndrome.

## 1. Introduction

Several classic studies have shown that GH deficiency (GHD) in adults is associated with abnormalities in body composition, metabolic derangements, and suboptimal physical performances; these impairments improve with GH replacement therapy [1–3]. Hypopituitarism is associated with increased cardiovascular mortality, women are disproportionally affected, and GH deficiency has been considered a contributory factor [4–6]. The increased mortality is reflected in elevated cardiovascular risk markers [7–9] and GH replacement results in improvement in these markers [10–14].

In obesity there is a markedly decreased GH secretion. For both children and adults, the greater the *body mass index *(*BMI*), the lower the GH response to provocative stimuli [15, 16], including the response to *GH-Releasing Hormone* (*GHRH*) [17, 18]. The altered somatotroph function of obesity is not permanent; it can be reversed by a return to normal weight [19] or by short-term calorie restriction [20]. The most striking example of reversibility appeared when obese subjects were treated with GHRH plus *GH-Releasing Peptide-*6 (*GHRP*-6) both at saturating doses, which resulted in a massive GH response for obese subjects [21]. This relative GH deficiency may contribute to develop or maintain the obese state [22] and GH treatment has been employed in obesity [23–26]. A recent meta-analysis of recombinant human GH as therapy for obesity in adults suggests that GH therapy leads to a decrease in visceral adiposity and increase in lean body mass as well as beneficial changes in lipid profile in obese adults, without inducing weight loss [25]. 

However, little is known about whether the association between decreased GH secretion and increased cardiovascular risk markers observed in patients with GHD and hypopituitarism occurs in obese patients without organic hypothalamic or pituitary disease. There are some recent interesting studies that have evaluated the relationship between GH secretion, evaluated by GHRH-arginine testing and cardiovascular risk markers or central adiposity measures. Makimura et al. found that the GH response to GHRH-arginine testing is reduced in both overweight and obese men and negatively associated with indices of central abdominal obesity including waist circumference, trunk fat, and visceral adipose tissue. They also found that the use of waist circumference adds predictive information to the determination of GH response, independent of BMI [27]. Utz et al. found that HDL was lower, high-sensitivity C-reactive protein (hsCRP) and *tumour necrosis factor*-*α* **(** *TNF-* *α* **)** receptor I higher, in young overweight or obese women meeting GHD criteria than in women with GH sufficiency. These differences remained significant after controlling for age plus BMI, waist circumference, or trunk fat; there were no differences in measures of insulin resistance [28]. Carmichael et al. found that peak GH response to GHRH-arginine, in a population of adults aged 50–90 years, was significantly related to fasting glucose, insulin, BMI, HDL cholesterol, triglycerides, trunk fat, and abdominal subregion fat, with fasting glucose ranking first by multiple regression analysis [29].

The most clearly altered GH secretion test in obesity is GHRH-induced GH secretion [18, 21, 30, 31], due to this markedly decreased GH secretion; it should be especially attractive to study its relationship with different cardiovascular risk factors in a homogenous population. We studied women of reproductive age because data in this group are particularly lacking, and it is very important to identify risk factors for future cardiovascular disease in this population. The aim of the present study was to evaluate the relationship between GHRH-induced GH secretion in obese premenopausal women and cardiovascular risk markers or insulin resistance.

## 2. Patients and Methods

### 2.1. Patients

Forty eight premenopausal obese patients, aged 35–52 years (32.6 ± 10.3 year), with a BMI of 36.0 ± 6.4 kg/m^2^, were studied (Table 1). None of the obese patients had diabetes mellitus or other medical problems nor were they taking any drugs. All subjects had regular menses. The subjects had been eating a weight-maintaining diet for several weeks prior to the study. *All the studies have been conducted in accordance with the declaration of Helsinki*. All subjects provided informed consent and approval for this study was obtained from the hospital committee.

### 2.2. Study Procedure

Between 08:30 and 09:00 AM, after an overnight fast and while seated, a peripheral venous line was obtained. Fifteen minutes later GHRH (100 micrograms, iv, 0 minutes) was administered. We obtained blood samples for GH at baseline (0 minutes) and then at times 15, 30, 60, 90, and 120 minutes. Fasting blood was drawn for IGF-I, serum cardiovascular risk markers, and hormonal determinations. Mid-waist circumference was measured as the midpoint between the iliac crest and the lowest rib, with the patient in the upright position. Measurement of the hip circumference was performed at the widest point, also with the subject in an upright position. Total body fat was calculated through bioelectrical impedance analysis (BIA). BIA is based on measurement of the transmission speed of a 1/4 volt electrical pulse between electrodes at the feet and hands. Because fat-free mass is comprised of water, proteins, and electrolytes, and conductivity is greater in fat-free mass than in fat mass [32]. Resistance and reactance are used to estimate total body water, and by extension, fat mass and lean mass, with the latter including bone [33]. Truncal fat was calculated through the Lemieux formula based on anthropometric parameters [34].

### 2.3. Assays and Other Methods

Serum samples were collected and stored at −80 C. Serum GH (*μ*g/L) was measured by a solid-phase, two-site chemiluminescent enzyme immunometric assay (Immulite, EURO/DPC) with a sensitivity of 0.01 *μ*g/L and with intrassay coefficients of variation of 5.3%, 6.0%, and 6.5% for low, medium, and high plasma GH levels, respectively; *and with interassay coefficients of variation of *6.5%, 5.5%,* and *6.6%* for low, medium, and high plasma GH levels, respectively*. IGF-I (ng/mL) was determined by a chemiluminescence assay (Nichols Institute, San Clemente, CA, USA) and with intrassay coefficients of variation of 4.8%, 5.2%, and 4.4% for low, medium and high plasma IGF-I levels, respectively, *and with interassay coefficients of variation of *7.7%, 7.4%, *and *4.7% *for low, medium, and high plasma IGF-I levels, respectively.* Insulin (*μ*U/mL) was measured with a solid-phase two-site chemiluminescent immunometric assay (Immulite 2000 Insulin, DPC, Los Angeles, CA, USA) and with intrassay coefficients of variation of 5.5%, 3.3%, and 3.7% for low, medium and high plasma insulin levels, respectively, *and with interassay coefficients of variation of *7.3%, 4.1%,* and *5.3%* for low, medium, and high plasma insulin levels, respectively.* Leptin (ng/mL) was measured by radioimmunoassay (Mediagnost, Tubigen, Germany) and with intrasay and interassay coefficients of variation of 5.3% and 13.6%, respectively. Glucose, total, HDL, and LDL cholesterol, triglycerides, glutamic oxalacetic transferase (GOT), glutamic pyruvic transferase (GPT), and gamma glutamyl transferase (GGT) were measured using standard laboratory previously described methods [35]. 

All samples from a given subject were analysed in the same assay run. The area under the secretory curve (AUC) was calculated by a trapezoidal method. Insulin resistance was calculated on the basis of fasting values of plasma glucose and insulin, according to the homeostasis model assessment (HOMA-IR) method [36] as follows: HOMA-IR = fasting insulin levels × fasting glucose levels/22.5, where basal insulin levels is in *μ*U/mL, and glucose is in mmol/L.

### 2.4. Statistical Analysis

The results are presented as mean values ± SEM. All comparisons were based on univariate, nonparametric tests. *Intragroup comparisons were based on Mann-Whitney test*. Numerical correlations were analyzed using Spearman's correlation test. *P* values ≤ .05 were considered to be significant. For graphic presentation we use mean values ± SEM. The SPSS software 12.0 (Chicago, IL, USA) was used to produce statistical analysis.

## 3. Results

### 3.1. Clinical Characteristics and Analytical Data of Study Subject

**Figure 1:**
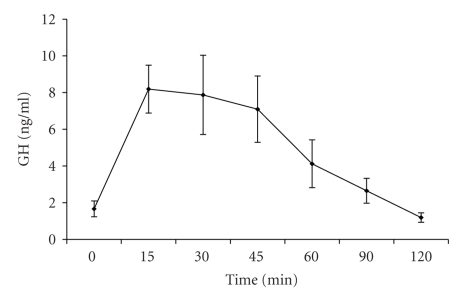
Mean ± SEM GH secretion in obese premenopausal women after stimulation with GHRH.

Clinical characteristics, cardiovascular risk markers, and hepatic aminotransferases are shown in Table 1. The age range of study participants was 32–48 years, and the BMI ranged from to 30.4 to 52.2 kg/m^2^. Subjects were arbitrarialy classified as meeting the criteria for GH deficiency (GHD) when peak GH after stimulation with GHRH was ≤3 ng/mL; although GHRH alone is not a well-validated test for the diagnosis of GH deficiency, this cutoff point is based on standard criteria used to diagnose adults with hypopituitarism after various stimuli [37, 38]. GH secretion after stimulation with GHRH at the different time points in the entire group is shown on Figure 1.

Clinical characteristics, cardiovascular risk markers, and hepatic aminotransferases in GH-Deficient and GH-Sufficient obese women are presented in Table 2. 23% of obese women met our stablished criteria for GHD based on the response to GHRH. The group of the patients classified as meeting hypopituitary GHD criteria had a mean BMI of 45.5 ± 6.3 kg/m^2^, with a midwaist circumference of 117 ± 9.2 cm, significantly higher than in obese patients with GH sufficiency (*P* = .007 for BMI and *P* = .039 for midwaist cicumference). The group of patients with peak GH after stimulation ≤3 *μ*g/L had a tendency to a higher mean age than the group of patients with peak GH after stimulation >3 *μ*g/L, 42.3 ± 9.0 versus 30.4 ± 9.4 , respectively, although the difference was not significant.

### 3.2. Relation of Peak-Stimulated GH to Measures of Adiposity

**Figure 2:**
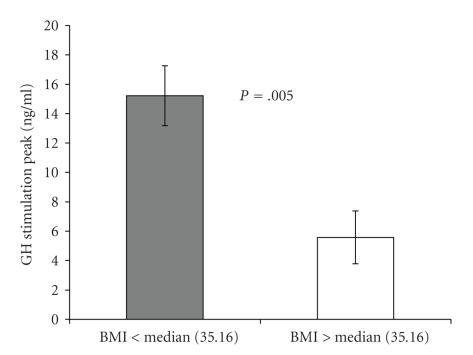
Mean ± SEM peak GH secretion in obese premenopausal women after stimulation with GHRH, divided in two groups, above or below BMI median (*P* = .005).

When the patients were divided in two groups, above or below BMI median, peak GH secretion was 15.2 ± 2.0 and 5.6 ± 1.8 (*μ*g/L) for patients above or below the median, respectively (*P* = .005) (Figure 2). BMI was strongly and inversely associated with peak GH after stimulation with GHRH (*R* = −0.721, *P* = .022). Peak GH secretion was strongly and inversely associated with waist circumference (*R* = −0.576, *P* = .020), (*R* = −0.748, *P* = .001), and trucal fat (*R* = −0.595, *P* = .019). These associations remained significant after controlling for age.

### 3.3. GH Secretion Parameters and Cardiovascular Risk Markers, Insulin Resistance Indices, and Hepatic Serum Aminotransferases

**Figure 3:**
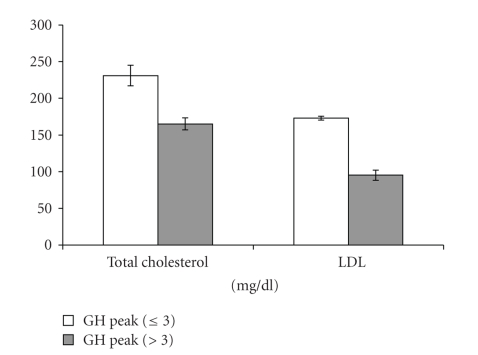
Mean ± SEM total and LDL cholesterol in GH-Deficient and GH-Sufficient Obese patients (*P* = .044).

**Figure 4:**
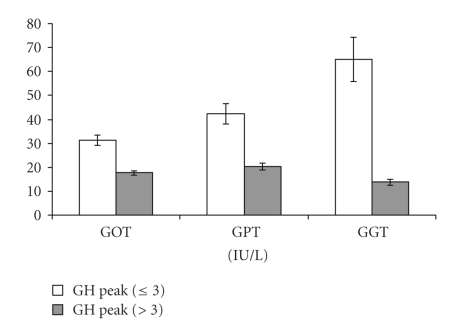
Mean ± SEM hepatic serum aminotransferases: GOT (*P* = .012), GPT (*P* = .022), and GGT (*P* = .007) in GH-Deficient and GH-Sufficient Obese patients.

**Figure 5:**
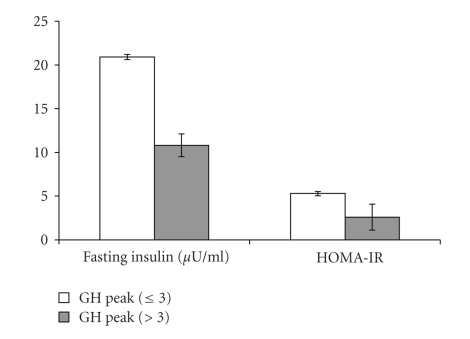
Mean ± SEM fasting insulin (*P* = .005) and HOMA-IR (*P* = .018) in GH-Deficient and GH-Sufficient Obese patients.

Mean levels of cardiovascular risk markers, hepatic aminotransferases, and insulin resistance were compared between in GHD and GH-Sufficient obese patients (Table 2). Mean total and LDL cholesterol were higher, in subjects who would have been classified as GHD based on hypopitutary criteria compared with GH-sufficient subjects (*P* = .039 and *P* = .044, resp.) (Figure 3). Mean serum hepatic aminotransferases, GOT, GPT, and GGT, were all higher, in subjects who would have been classified as GHD based on hypopitutary criteria when compared with GH-sufficient subjects (*P* = .012, *P* = .022, and *P* = .007, resp.) (Figure 4). Mean fasting insulin and HOMA-IR were higher, in the group of patients with peak GH after stimulation ≤3 *μ*g/L when compared with the group of patients with peak GH after stimulation >3 *μ*g/L (*P* = .005 and *P* = .018, resp.) (Figure 5). These differences were no longer significant after controlling for measures of adiposity whether BMI, mid-waist circumference, or trunk fat.

Correlations between GH secretion or IGF-I and cardiovascular risk markers or aminotransferases are shown in Table 3. Peak GH secretion after stimulation with GHRH was inversely associated with total (*R* = −0.532, *P* = .034) and LDL cholesterol (*R* = −0.692, *P* = .006) and positively associated with HDL cholesterol (*R* = 0.561, *P* = .037). Peak GH secretion after stimulation with GHRH was inversely associated with serum hepatic aminotransferase, GOT (*R* = −0.685, *P* = .020), GPT (*R* = −0.656, *P* = .011), and GGT (*R* = −0.642, *P* = .018). Peak GH secretion after stimulation with GHRH was inversely associated with fasting insulin (*R* = −0.650, *P* = .012) and HOMA-IR (*R* = −0.846, *P* = .001). These correlations were no longer significant after controlling for measures of adiposity whether BMI, mid-waist circumference, or trunk fat.

IGF-I was strongly and inversely associated with BMI (*R* = −0.577, *P* = .024) and percentage of total body fat (*R* = −0.728, *P* = .002). IGF-I was strongly negatively associated with LDL cholesterol (*R* = −0.725, *P* = .005) and positively associated with HDL-cholesterol (*R* = 0.568, *P* = .043). IGF-I was strongly negatively associated with HOMA-IR (*R* = −0.613, *P* = .034). These correlations were no longer significant after controlling for measures of adiposity whether BMI, mid-waist circumference, or trunk fat. HOMA-IR was strongly and positively associated with serum hepatic aminotransferases, GOT (*R* = 0.821, *P* = .023), GPT (*R* = 0.705, *P* = .023), and GGT (*R* = 0.693, *P* = .026).

## 4. Discussion

We have found a strong inverse relationship between BMI or indices of central adiposity and peak-stimulated GH levels. The presence of decreased GH secretion is associated with a deleterious cardiovascular marker risk profile, increases in total and LDL cholesterol. We report strong inverse associations of GH peak after stimulation by GHRH in a group of obese otherwise healthy premenopausal women with traditional lipoprotein cardiovascular risk markers. Our results are similar to those of Utz et al. [28], although they found significant differences with other cardiovascular risk factors, mainly nontraditional, in that study HDL was lower, hsCRP (high-sensitivity C-reactive protein), and TNF-*α* receptor I higher, in young overweight or obese women meeting GHD criteria than in women with GH sufficiency. In contrast to Utz et al. data, in this study the increase in total and LDL cholesterol was no longer significant after controlling for measures of adiposity whether BMI, mid-waist circumference, or trunk fat. These differences could be due in part to the different stimulus employed or to the different patient group; we include only obese women without other disease, or to the use of indirect indices of visceral fat. We think that all these data raise the question of whether decreased endogenous GH secretion may contribute to an elevated risk of future cardiovascular events in young obese women.

The primary cause of the impaired GH secretion of obesity could be an altered hypothalamus, abnormal pituitary function, or a perturbation of the peripheral signals acting at either the pituitary or hypothalamic level. There are different studies that suggest that the pathophysiological mechanism responsible for the GH hyposecretion of obesity is probably multifactorial; there is a chronic state of somatostatin hypersecretion, increased FFA and decreased ghrelin [39–44]. Quite surprisingly, we found in the present study that decreased GH secretion was associated with increased levels of serum hepatic aminotransferases. We report a strong inverse association of GH peak after stimulation by GHRH in a group of obese otherwise healthy premenopausal women with *increased* hepatic aminotransferases. As far as we know, there has not been previously published such a relationship. Although there are some recent data that suggest that GH deficiency could participate in the pathogenesis of nonalcoholic fatty liver disease [45]. We hypothesize that the mechanism of that correlation is a manifestation of hepatic insulin resistance. It has been found that the higher the BMI, the higher the prevalence of elevated liver enzymes, and the presence of insulin resistance was highly predictive of elevated hepatic enzymes, suggesting an important role for insulin resistance in nonalcoholic fatty liver disease [46]. In our study HOMA-IR was strongly and positively associated with serum hepatic aminotransferases. The presence of decreased GH secretion is associated with increased serum insulin levels and insulin resistance indices. Moreover, we report a strong inverse association of GH peak after stimulation with GHRH and the insulin resistance indices, HOMA-IR. The importance of fasting insulin in GH secretion has been found in some studies [29, 47]. There is also clinical [48] and experimental [49] evidence suggesting an important role for insulin as a direct inhibitor of GH secretion. All these data suggest that insulin resistance could be another factor responsible for the altered GH secretion of obesity. 

In accordance with previous studies [50, 51] we have found that in the presence of decreased GH secretion, IGF-I levels were normal, although IGF-I was positively associated with peak GH secretion. But IGF-I was strongly and inversely associated with BMI and insulin resistance (HOMA-IR). These data suggest, on one hand, that IGF-I is still GH dependent in obesity, but that there are other factors such as insulin or others [51] that regulate IGF-I levels. On the other hand that the decreased GH secretion of obesity is not due to increased IGF-I levels as previously suggested [52].

There are many common features between subjects with GHD due to pituitary disease and subjects with a clustering of cardiovascular risk factors in the absence of pituitary disease, but it is not clear whether the low GH associated with these cardiovascular risk factors is cause or effect. Low GH in the setting of obesity has been shown to be reversed with weight reduction, implying that it is obesity that causes the low GH [19, 30]. By contrast, the observation that subjects with GHD due to pituitary or hypothalamic disease develop obesity and its metabolic consequences [1–3], and that some cardiovascular risk factors are inversely related to GH independently of obesity indices [28], suggests that GH plays a physiological role in the development of obesity and its cardiovascular complications. This distinction is important in our understanding of the role of GH in the development and maintenance of cardiovascular risk factors and may have therapeutic implications.

Although our data do not differentiate between low GH being a cause or an effect of these cardiovascular risk factors, they indicate that the relationship between low GH and increased cardiovascular risk may be physiologically important in the absence of pituitary disease. Another limitation that should be considered in this study is the lack of a control lean group. These data in a group of obese young women showing a clear correlation of low GH to individual cardiovascular risk factors suggest an important role for GH in the clustering of these risk factors, whether or not the relationship is a causative one. Analogous to the gradual acceptance of a wide variety of what are now considered traditional cardiovascular risk factors, perhaps it is time to consider low GH as an independent marker for cardiovascular disease [29].

## 5. Conclusions

In conclusion, we have found that obese premenopausal women with decreased GHRH-induced GH secretion when compared with obese patients with GH sufficiency, have increased total cholesterol, LDL cholesterol, liver enzymes, fasting insulin and HOMA-IR, when compared with obese patients with GH sufficiency. There was a significant negative correlation between peak GH secretion and total cholesterol, LDL cholesterol, liver enzymes, fasting insulin or HOMA-IR. There was a significant positive correlation between IGF-I and peak GH and a negative correlation between IGF and HOMA-IR, but IGF-I levels were not decreased in the GHD group. We think that these data strongly suggest a role for insulin resistance in the decreased GH secretion of obesity, and the concept that the blunted GH secretion of central obesity could be the pituitary expression of the insulin resistance (metabolic) syndrome.

## Figures and Tables

**Table 1:** Clinical characteristics, cardiovascular risk markers, and hepatic aminotransferases in obese premenopausal women (*n* = 48).

	Mean ± SEM
Age (year)	32.6 ± 2.6
BMI (kg/m^2^)	36.0 ± 1.6
Mid-waist circumference (cm)	102.4 ± 3.4
Waist/hip ratio	0.90 ± 0.02
Total body fat (%)	38.7 ± 1.5
Trunk fat (cm^2^)	141.9 ± 10.2
GH Peak (ng/mL)	10.4 ± 1.8
IGF-I (ng/mL)	147.3 ± 69.0
Total cholesterol (mg/dL)	177.1 ± 11.2
HDL (mg/dL)	52.6 ± 2.0
LDL (mg/dL)	106.3 ± 9.6
Triglyceride (mg/dL**)**	102.4 ± 18.3
Fasting glucose (mg/dL)	95.7 ± 2.0
Fasting Insulin (*μ*U/mL)	13.0 ± 0.5
HOMA-IR	3.3 ± 0.5
TAS	122.5 ± 3.5
TAD	76.5 ± 3.2
Leptin (ng/mL)	24.3 ± 13.5
GOT (IU/L)	21.4 ± 8.0
GPT (mIU/L)	24.9 ± 12.7
GGT (IU/L)	25.6 ± 27.3

**Table 2:** Cardiovascular risk markers and hepatic aminotransferases (Mean ± SEM) in GH-Deficient and GH-Sufficient Obese premenopausal women.

	GH Peak (≤3 ng/mL)	GH Peak (>3 ng/mL)	*P* value
	*n* = 9	*N* = 39	
Age (year)	42.3 ± 2.3	30.4 ± 2.4	NS
BMI (kg/m^2^)	45.5 ± 1.6	33.8 ± 1.0	.007
Mid-waist circumference (cm)	117 ± 2.3	99.0 ± 3.1	.039
Waist/hip ratio	0.97 ± 0.04	0.89 ± 0.02	NS
Total body fat (%)	46.9 ± 1.0	36.8 ± 1.2	.004
Trunk fat (cm^2^)	185.0 ± 6.7	131.2 ± 9.2	.048
IGF-I (ng/mL)	103.3 ± 6.5	158.3 ± 18.2	NS
IGFBP-3	4.9 ± 0.2	4.6 ± 0.3	NS
Total cholesterol (mg/dL)	231 ± 14.0	165 ± 8.3	.039
HDL (mg/dL)	43.5 ± 2.0	54.2 ± 1.8	NS
LDL (mg/dL)	173 ± 2.5	95.2 ± 7	.044
Triglyceride (mg/dL**)**	172 ± 29.5	86.4 ± 13.4	.047
Fasting glucose (mg/dL)	99.7 ± 2.0	91.7 ± 2.0	NS
Fasting Insulin (*μ*U/mL)	20.9 ± 0.3	10.8 ± 1.3	.005
HOMA-IR	5.3 ± 0.25	2.6 ± 1.5	.018
Leptin	20.1 ± 3.5	24.7 ± 3.6	NS
GOT (IU/L)	31.3 ± 2.0	17.6 ± 1.0	.012
GPT (IU/L)	42.3 ± 4.4	20.2 ± 1.5	.022
GGT (IU/L)	65.0 ± 9.2	13.8 ± 1.2	.007

**Table 3:** Correlations between GH secretion or IGF-I and cardiovascular risk markers or aminotransferases levels.

	GH Peak		IGF-I	
	*r*	*P*	*r*	*P*
Age (year)	−0.543	.030	−0.486	.066
BMI (kg/m^2^)	−0.721	.022	−0.577	.024
Mid-waist circumference (cm)	−0.576	.020	−0.338	.218
Waist/hip ratio	−0.350	.184	−0.229	.411
Total body fat (%)	−0.748	.001	−0.728	.002
Trunk fat (cm^2^)	−0.595	.019	−0.338	.218
GH Peak			0.599	.018
IGF-I (ng/mL)	0.599	.018		
Total cholesterol (mg/dL)	−0.532	.034	−0.513	.051
HDL (mg/dL)	0.561	.037	0.568	.043
LDL (mg/dL)	−0.692	.006	−0.725	.005
Triglyceride (mg/dL)	−0.456	.076	−0.468	.078
Fasting Insulin (*μ*U/mL)	−0.650	.012	−0.506	.078
HOMA-IR	−0.846	.001	−0.613	.034
GOT (IU/L)	−0.685	.020	−0.049	.894
GPT (IU/L)	−0.656	.011	−0.257	.397
GGT (IU/L)	−0.642	.018	−0.408	.188
